# Genomic Surveillance of Recent Dengue Outbreaks in Colombo, Sri Lanka

**DOI:** 10.3390/v15071408

**Published:** 2023-06-21

**Authors:** Sachith Maduranga, Braulio Mark Valencia, Chathurani Sigera, Thiruni Adikari, Praveen Weeratunga, Deepika Fernando, Senaka Rajapakse, Andrew R. Lloyd, Rowena A. Bull, Chaturaka Rodrigo

**Affiliations:** 1 School of Biomedical Sciences, UNSW Sydney, Sydney, NSW 2052, Australia; sarachchige@kirby.unsw.edu.au (S.M.); tadikari@kirby.unsw.edu.au (T.A.); r.bull@unsw.edu.au (R.A.B.); 2 Kirby Institute, UNSW Sydney, Sydney, NSW 2052, Australia; barroyo@kirby.unsw.edu.au (B.M.V.); a.lloyd@unsw.edu.au (A.R.L.); 3 Faculty of Medicine, University of Colombo, Colombo 00800, Sri Lanka; chathuranisigera@med.cmb.ac.lk (C.S.); praveen@clinmed.cmb.ac.lk (P.W.); deepika@parasit.cmb.ac.lk (D.F.); senaka@med.cmb.ac.lk (S.R.)

**Keywords:** dengue, Sri Lanka, phylogenetics, phylogeography, serotype, genotype, epidemiology

## Abstract

All four serotypes of the dengue virus (DENV1–4) cause a phenotypically similar illness, but serial infections from different serotypes increase the risk of severe disease. Thus, genomic surveillance of circulating viruses is important to detect serotype switches that precede community outbreaks of disproportionate magnitude. A phylogenetic analysis was conducted on near full length DENV genomes sequenced from serum collected from a prospective cohort study from the Colombo district, Sri Lanka during a 28-month period using Oxford nanopore technology, and the consensus sequences were analyzed using maximum likelihood and Bayesian evolutionary analysis. From 523 patients, 328 DENV sequences were successfully generated (DENV1: 43, DENV2: 219, DENV3:66). Most circulating sequences originated from a common ancestor that was estimated to have existed from around 2010 for DENV2 and around 2015/2016 for DENV1 and DENV3. Four distinct outbreaks coinciding with monsoon rain seasons were identified during the observation period mostly driven by DENV2 cosmopolitan genotype, except for a large outbreak in 2019 contributed by DENV3 genotype I. This serotype switch did not result in a more clinically severe illness. Phylogeographic analyses showed that all outbreaks started within Colombo city and then spread to the rest of the district. In 2019, DENV3 genotype I, previously, rarely reported in Sri Lanka, is likely to have contributed to a disease outbreak. However, this did not result in more severe disease in those infected, probably due to pre-existing DENV3 immunity in the community. Targeted vector control within Colombo city before anticipated seasonal outbreaks may help to limit the geographic spread of outbreaks.

## 1. Introduction

Dengue fever is a mosquito borne flavivirus infection categorized as a neglected tropical disease by the World Health Organization [1]. It is estimated that 390 million dengue infections occur annually, of which 96 million are clinically apparent [2]. Endemic disease transmission occurs in at least 129 countries [3], and those with the highest disease burden are low- to middle-income countries with limited resources for patient care and scientific study of the disease. Dengue infection in humans is caused by a sero-complex of related flaviviruses DENV1, DENV2, DENV3 and DENV4 (serotypes). Phylogenetic analysis has further subcategorized each serotype into distinct genotypes isolated from various geographical areas [4]. In the dengue literature, serotypes are given more prominence than genotypes, as they are closely linked to case incidence and clinically observed disease severity. 

Clinical manifestations of dengue range from asymptomatic and sub-clinical infections to uncomplicated dengue fever, to life threatening severe dengue/dengue hemorrhagic fever [5,6]. Prior infection with dengue is a key risk factor for complications during a subsequent infection due to antibody dependent enhancement. That is, antibodies formed against one serotype are capable of binding to but not neutralizing a second serotype, triggering an abnormal and exaggerated inflammatory response [7,8]. Epidemiological evidence supports this theory, as severe waves of infection are observed when there is a change in the circulating serotype in the community as detected by virological surveillance [9,10]. As the mosquito vectors (*Aedes aegypti* and *Aedes albopictus*) are abundant in many countries [11], combined with cross border human movement, this has resulted in a dramatic increase in the last 50 years; switches in circulating serotypes are frequently observed. This increases the risk of individuals (particularly in younger age groups) having severe infection. Phylogenetics based surveillance of circulating serotypes/genotypes is useful to predict outbreaks of severe disease, but the associated costs of large-scale viral sequencing are beyond the capacity of most non-high-income countries unless done as part of a research project. 

The airborne range of the dengue vector mosquito is limited with a mean distance travelled of only around 100 m from their breeding area [12]. However, during an outbreak, the number of cases increase rapidly across a vast geographic area, usually within weeks [13]. Thus, when the vector mosquito is endemic and ubiquitous, movement of infected humans may be the primary driver for geographic spread of an outbreak. Phylogenetic analysis (of viral genomes) when combined with spatio-temporal localization (phylogeography) is a powerful tool that can provide insights into this hypothesis. If distinct dengue outbreaks within a geographic area can be defined and if viral sequences and their location of isolation during such outbreaks are known, phylogeography can identify the likely origin (location) of these outbreaks. This is important to understand the transmission dynamics of the virus [14,15] in a locality, which in turn may help outbreak control via targeted vector control in hotspots for transmission. 

In this study, combined phylogenetic and phylogeography analysis of viral sequences isolated within the Colombo district of Sri Lanka, we aimed to: (a) characterize the phylogeny of circulating DENV over a time period from October 2017–January 2020, (b) explore the likely geographical origin of outbreaks identified during this period, and (c) observe if a shift in the dominant circulating serotype or genotype was associated with an increased clinical severity of disease.

## 2. Materials and Methods

### 2.1. Clinical Samples

The human plasma samples for this analysis were sourced from the Colombo Dengue Study (CDS) [16,17]. CDS is an ongoing prospective cohort study (active since 2017) in Sri Lanka: a tropical island in the Indian Ocean where an estimated 22 million people inhabit an area of 65,000 km_2_. The country is divided into 25 administrative districts and dengue cases are reported from all of them as the vector mosquitoes (*Aedes* sp.) are found throughout the island. CDS recruits patients clinically suspected of having dengue fever from the National Hospital of Sri Lanka in Colombo (city) which is the largest tertiary level hospital in Sri Lanka. The details of CDS (methods, data on clinical parameters and demographics) have been published previously [16,17]. In brief, the primary purpose of the cohort is to identify early risk associations for dengue associated plasma leakage [18]. Thus, patients presenting within the first 96 h of fever and who were clinically suspected of having dengue were recruited and followed up daily to record adverse outcomes including plasma leakage and shock. Dengue infection was confirmed by both NS1 antigen testing in real time and RT-PCR (conducted retrospectively on stored sera), enabling a nested case–control comparison between those with dengue fever and others, as well as dengue patients with and without plasma leakage. Demographic information including the postcode of residence was collected from all patients recruited to CDS. Only samples collected during the first phase of CDS between October 2017 and January 2020 were included in this analysis. 

**Figure 1:**
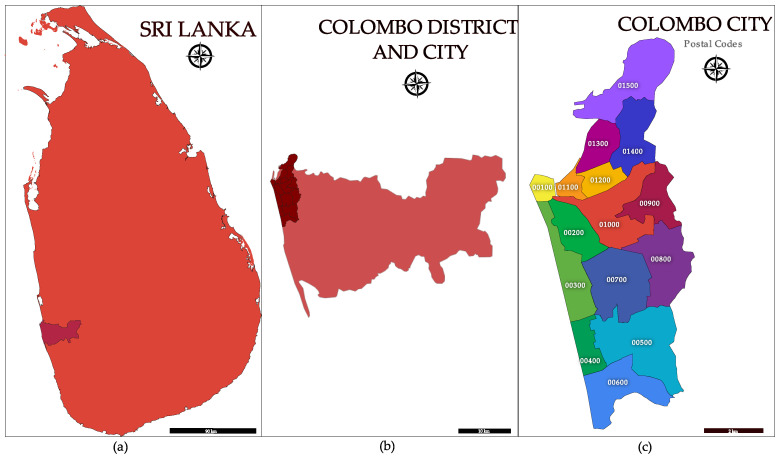
Panel (**a**)—Colombo district within Sri Lanka, Panel (**b**)—Colombo city (shaded dark red) within the Colombo district, Panel (**c**)—Postcodes 00100—01500 of Colombo city.

The Colombo city, the largest metropolitan area of Sri Lanka is located within the district of Colombo (Figure 1). Since both the city and the district have the same name, to avoid confusion, this paper will highlight whether the district or city is referred to at each mention of “Colombo”. The Colombo district is the smallest out of all administrative districts in Sri Lanka with an area of 699 km^2^ (1.1% of total land area) [19], but it also has the highest population with an estimated 2.47 million people (11% of the total Sri Lankan population) [20]. Hence, population density is highest within this district. In addition to Colombo city, which is the commercial capital of Sri Lanka, the Colombo district also contains the administrative capital of Sri Lanka, and the port of Colombo. The main international airport of Sri Lanka is in the neighboring Gampaha district approximately 32 km away by road from the Colombo city center. In addition to the resident population of the district, approximately 1 million people commute daily from the neighboring districts for work and education. The area of the Colombo district is further subdivided into 58 postcodes noted by a 5-digit number and Colombo city is denoted by the first 15 postcodes from 00100–01500 (Figure 1) [21]. Colombo city is even more congested than the rest of the Colombo district with approximately 633,000 people living in an area of 37.3 km^2^ in 2023 [22]. 

### 2.2. Viral Sequence Generation

CDS is maintained in a collaboration between University of Colombo, Sri Lanka, and University of New South Wales (UNSW), Sydney, Australia. Plasma samples collected on admission are shipped to the Kirby institute, UNSW, Sydney for viral genome extraction and sequencing. Near full-length DENV genomes were reverse transcribed and amplified as whole intact molecules with a nested PCR. Sequencing by Oxford Nanopore Technology (ONT) was performed at the Kinghorn Center for Clinical Genomics, Sydney on a GridION sequencer using a FLO-MIN107 v9.5 flow cell with ligation barcoding (ONT ligation sequencing kit SQK-LSK109). Signal-level data from the sequencer was base-called, filtered for high-quality reads (mean Q-score > 7) and demultiplexed with Guppy (version 2.3.5 and 3.0.3, http://staff.aist.go.jp/yutaka.ueno/guppy/, accessed on 10 October 2022), and further processed with the Nanopolish algorithm (version 0.11.1, https://github.com/jts/nanopolish, accessed on 15 October 2022). The details of DENV full genome amplification assay (including primers used for each serotype), the specifics of ONT sequencing and the associated data analysis pipeline, to generate consensus sequences, has been published previously [23]. The accuracy of DENV consensus sequences generated by this method were comparable to those generated by second generation paired-end short read (Illumina) technology [23].

### 2.3. Phylogenetic Analysis

The serotype and genotype of DENV sequences were identified by comparing against the Genbank database (BLAST-n) and reconfirmed by an independent Dengue Virus Typing Tool (https://www.genomedetective.com/app/typingtool/dengue/, accessed on 2 November 2022) [24,25]. To describe the general phylogeny, all available DENV sequences from CDS were aligned with MUltiple Sequence Comparison by the Log-Expectation algorithm (MUSCLE, v3.7) [26], and a maximum likelihood of phylogenetic trees were made with the Randomized Axelerated Maximum Likelihood tool (RAXML) [27] implemented within the CIPRES Science Gateway [28], as the consensus of a 1000 bootstrap replicates. Only nodes with a bootstrap support >70% were visualized in this consensus tree.

The phylodynamic analyses were performed with the Bayesian Evolutionary Analysis by Sampling Trees (BEAST) software suite (v1.10). The multiple sequence alignments were exported to the BEAUti software (v1.10.4) to generate the input .xml file to be run with BEAST. To identify the best nucleotide substitution model (TN93, HKY, GTR) and clock model (strict clock, uncorrelated relaxed lognormal clock, uncorrelated relaxed exponential clock) that fits the available data, a model selection was conducted by running randomly selected 50 serotype-specific sequences from CDS (3 alignments, one per serotype) in BEAST to test different combinations of above models in a 3 × 3 matrix using path and stepping-stone sampling. The Monte Carlo Markov chain (MCMC) implemented within BEAST to identify the best fitting phylogenic parameters were run for a total length of 10^9^ states with sampling at every 10^5^ states. The MCMC was considered to have reached an equilibrium (optimum parameter values identified) when the effective sampling size (ESS) was greater than 200 estimates. Output of each BEAST run was visualized with the Tracer software (v1.7.2) after discarding the first 10% of estimates from the MCMC (burn-in). The maximum clade credibility (MCC) tree from the BEAST output was generated using the TreeAnnotator (v1.10.4) tool with a 10% burn-in, collapsing the nodes with a posterior probability <0.5 and was visualized with Figtree software (v1.4.2). Each BEAST analysis was run on the CIPRES Science Gateway (version 3.3, https://www.phylo.org/, accessed on 15 November 2022) [28] using the high-performance BEAGLE library. 

Once the best fitting substitution and clock models were identified, multiple sequence alignments for the whole dataset or its subsets (as described for the phylogeography analysis below) were imported into BEAST to generate MCC trees using selected models. The first analysis aimed to describe the general phylogeny of all DENV sequences from CDS to identify ancestral nodes for currently circulating variants (supplementing the maximum likelihood trees described above). Subsequent analyses described in the next two sections were limited to sequences isolated from people resident within the Colombo district. 

### 2.4. Identification of Sequence Clusters from Distinct Outbreaks

Since DENV infection is endemic in Sri Lanka throughout the year, identifying distinct outbreaks is difficult. Therefore, a two-step approach, initially based on epidemiological surveillance, and subsequently on the analysis of viral sequence phylogenies, was used.

Dengue is a notifiable disease in Sri Lanka and all clinically suspected cases must be reported to the epidemiology unit of Ministry of Health in Sri Lanka. This data is publicly available. However, RT-PCR is not routinely performed for diagnosis; therefore, serotype data is unavailable from this source. Monthly dengue case numbers reported from September 2017 to January 2020 in Colombo district were superimposed on the 10-year mean (2010–2020) of the same parameter. Patterns on these two traces were used to identify the timings of distinct seasonal outbreaks. These time periods are henceforth referred to as “temporal outbreak bins”. 

Next, CDS sequences from each temporal outbreak bin, obtained from samples collected from individual’s resident within Colombo district postcodes, were compiled into genotype specific alignments with MUSCLE v3.7, if there were more than 10 sequences per alignment. MCC trees were generated with BEAST v1.10 as described above (MCMC length: 10^9^ states, sampling every 10^5^ states). Using the time axis of the MCC tree, sequences emerging from a most recent common ancestor that existed up to 18 months prior to the start of the temporal outbreak bin were selected as sequences of that outbreak. An 18-month window was allowed as the determinations of ancestral node timing are not precise and have an associated high-posterior density interval. 

### 2.5. Phylogeography Analysis

The sequences from a distinct outbreak, identified using the two-step approach described above, were compiled into genotype specific alignments and run in BEAST (MCMC length: 10^9^ states, sampling every 10^5^ states) with the postcode of residence of the subject (5-digit number) mapped as a trait to the phylogeny [29]. The MCC trees from these runs output the likely location (postcode) of the ancestral sequences enabling an estimation of the location of origin of each cluster. 

### 2.6. Correlation of Serotype Switches with Disease Severity

When a likely outbreak event was dominated by a serotype different to that of previous outbreaks, we compared the incidence of severe dengue, plasma leakage, symptom severity and duration of illness (date of discharge from hospital subtracted by the self-reported date of symptom onset) between the outbreaks to identify statistically significant differences. Severe dengue was defined according to the WHO’s 2009 clinical classification [5]. Plasma leakage was identified when there was a >20% rise in hematocrit compared to baseline, or when an absolute hematocrit reading was >45%, or when fluid accumulation was demonstrated by ultrasonography within pleural or peritoneal cavities. A symptom severity score was defined by assigning equal weightage (0 = absent, 1 = present) to 13 daily recorded symptoms (abdominal pain, arthralgia, cough, chills or rigors, difficulty in breathing, headache, fainting, loss of appetite, backache, myalgia, nausea or vomiting, retro-orbital pain, vertigo) and taking the daily maximum score per patient during hospitalization. Logistic (for discrete outcomes) or linear (for continuous outcomes) regressions were conducted to identify statistically significant associations adjusting for prior dengue infection. Prior dengue infection was determined by the presence anti-DENV envelope IgG in the baseline sample (within 3 days of onset) as measured by ELISA (Euroimmune, Lubeck, Germany) [30]. Since plasma was collected within 96 h at the onset of fever, the presence of anti-DENV IgG is likely due to prior infection. Statistical significance was set at *p* < 0.05. 

## 3. Results

### 3.1. Viral Sequences and Phylogeny

**Figure 2:**
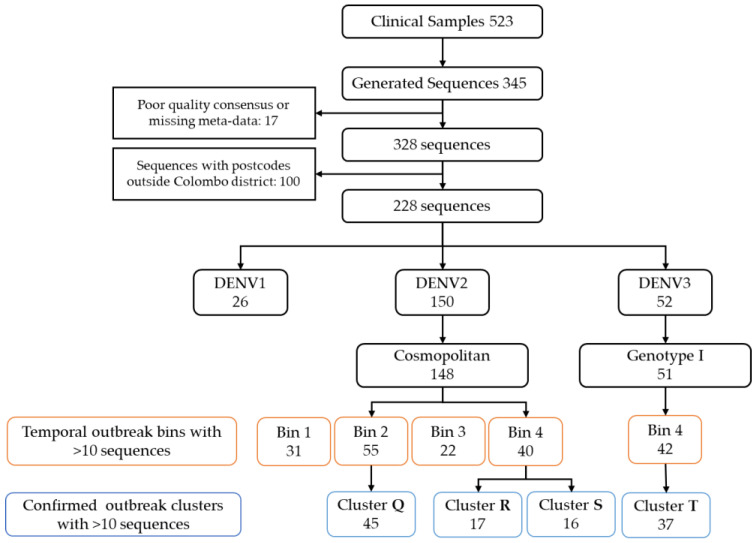
Flowchart showing sequence selection for each component of analysis. General phylogenies were made with 328 sequences meeting quality control requirements. Outbreak and phylogeography analyses were performed with sequences isolated from people resident within the Colombo district (*n* = 228).

**Figure 3:**
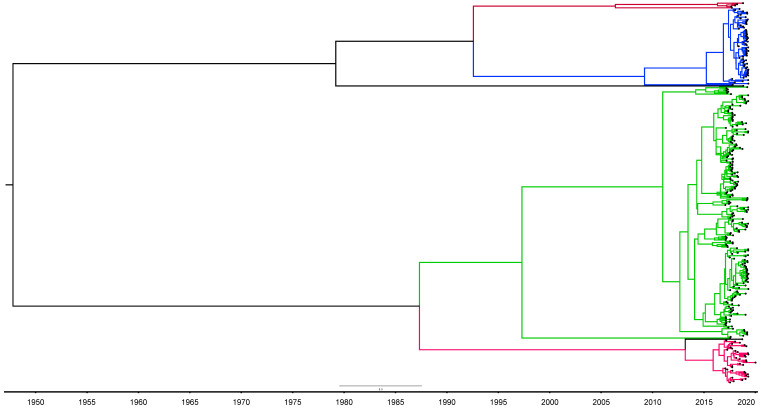
Maximum Clade Credibility tree generated from Bayesian evolutionary analysis (BEAST) with nodes collapsed to show only those with posterior probability >0.5. DENV1—red, DENV2—green, DENV3—blue. The timeline is in calendar years.

A total of 345 near-full-length DENV consensus sequences were generated from 523 viremic samples (one per patient) collected within CDS from 11 October 2017 to 1 February 2020. Extraction of genomes from remaining samples most likely failed due to low viral loads. Median viral loads (and interquartile range) of samples that were successfully amplified or not were 22688 (IQR: 4090–934114) PFU/mL and 6571 (IQR: 1903–78343), respectively (*p* < 0.02, Kruskal–Wallis Test). Of the 345 available genomes, 17 sequences were removed due to lack of clinical and epidemiological metadata or poor quality of the consensus sequences preventing reliable serotype/genotype classification. For analyses related to outbreaks, 100 further sequences linked to postcodes outside of the Colombo district were also excluded. A flow chart of sequence selection at each step of the analysis is shown in Figure 2. The serotype and genotype breakdown of the whole CDS dataset of DENV sequences is provided in Table 1. The model selection process, prior to Bayesian phylogenetic analysis, did not reveal a significantly better performance (via Bayes factor calculation) by any single model or their combinations for any of the serotypes. Given the comparable performance of all models, the lognormal relaxed clock model and GTR substitution model were arbitrarily selected for all subsequent analyses. The phylogeny of all CDS sequences were visualized as timed phylogenetic trees generated via Bayesian evolutionary analysis (maximum clade credibility tree with nodes of posterior probability >0.5 shown, Figure 3) and also as a maximum likelihood (ML) phylogenetic tree (consensus of X1000 bootstrap replicates with >70% bootstrap support per node, Supplementary Figure S1). The ML tree was built with near-full-length genomes, while the Bayesian phylogeny was built using part of the genome coding for structural proteins only (capsid-prM-envelope). The topology of both trees was similar.

Both trees showed predominance of the DENV2 cosmopolitan genotype throughout the period of observation. Two DENV1 genotypes were noted with one confirmed as DENV1 genotype I. For the others, BLAST results (DENV1 genotype V) and those of the genotyping tool (DENV1 unclassified but related to genotype III) showed a conflict and these were kept as DENV1 uncategorized rather than assigning a genotype. For DENV3, the majority of the sequences belonged to genotype I and these were identified after mid-2019. DENV3 genotype I had only been identified from two samples previously in Sri Lanka once in 2004 and later in 2017. To further explore this unexpected finding, we downloaded all publicly available annotated DENV3 genotype I sequences longer than 1000nts and isolated from human samples (*n* = 627) from bacterial and viral bioinformatics resource center (BV-BRC, https://www.bv-brc.org/, accessed on 12 March 2023) [30] and aligned these with our DENV3 genotype I sequences to generate a maximum likelihood phylogenetic tree (consensus of 1000 bootstrap trees). Sequences identified in Indonesia and Taiwan around 2015–2016 had the closest phylogenetic similarity to our sequences (Supplementary Figure S3). 

The Bayesian phylogenetic analysis showed that a majority of extant circulating sequences within the same genotype could be traced to a recent common ancestor that existed up to 10 years in the past. For the DENV2 sequences, this ancestor existed around 2010, while for DENV1 and DENV3, the ancestors existed around 2015–2016. However, a few other sequences from the same serotypes and genotypes, which originated from an ancestor further back in the timeline, probably represent lineages from prior outbreaks that were phased out by recent dominant lineages. The timing of the DENV3 genotype I ancestor in Sri Lanka (circa 2015), observed from the Bayesian MCC tree, matches with the observation of the ML tree of the global DENV3 genotype I sequences, in that, our sequences had the most phylogenetic similarity with Indonesian and Taiwanese sequences isolated during 2015/2016. DENV3 genotype I sequences isolated from the same countries after 2015/2016 were phylogenetically more distant (Supplementary Figure S3). 

### 3.2. Identification of Distinct Outbreaks

**Figure 4:**
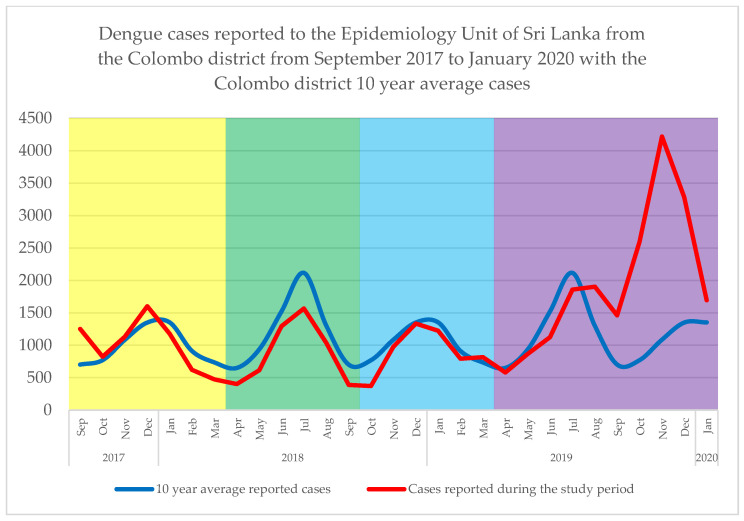
Monthly dengue case numbers reported (Source: Epidemiology Unit, Ministry of Health, Sri Lanka) from September 2017 to January 2020 (**red**) superimposed on the 10-year (2010–2020) mean of the same parameter (**blue**). The temporal outbreak bins are shaded in yellow, green, pale blue and purple, respectively.

The monthly number of dengue cases reported to the Epidemiology unit of the Ministry of Health, Sri Lanka [31], from the Colombo district from September 2017 to January 2020 (in red), were superimposed on the average monthly case numbers between 2010–2020 (in blue), as seen in Figure 4. This clearly shows identifiable patterns of distinct outbreak events peaking between November to January and June to August in each year, corresponding to the two monsoon rain seasons. With increased rainfall, the mosquito breeding sites are expected to increase [13]. However, from mid-2019 onwards, an outbreak of unusual magnitude was observed, which seemed to merge onto the first monsoon-driven outbreak of the same year. Hence, it was considered here as a single extended outbreak. Overall, four temporal outbreaks bins were defined based on the observations as follows: (a) September 2017 to March 2018, (b) April 2018 to September 2018, (c) October 2018 to March 2019, and (d) April 2019 to January 2020.

Sequences from the same genotype which originated from postcodes within the Colombo district were classified according to temporal outbreak bins only; serotypes 2 and 3 had >10 sequences per bin for further evaluation. This included DENV2 (cosmopolitan genotype) sequences for all four bins, and the DENV3 genotype I sequences for bin 4. After generating Bayesian phylogenies (MCC trees using near-full-length genomes) for these five datasets, four refined clusters with >10 sequences attributable to a distinct outbreak within a temporal bin were identified (Supplementary Figure S2A–E). These epidemiologically and phylogenetically confirmed outbreak clusters were renamed as Q, R, S and T (Table 2). Cluster Q was from temporal bin 2 and had the DENV2 cosmopolitan genotype. Clusters R and S were both from temporal bin 4 and had the DENV2 cosmopolitan genotype. Cluster T was from temporal bin 4 and had DENV3 genotype I. 

### 3.3. Phylogeography Analysis

**Figure 5:**
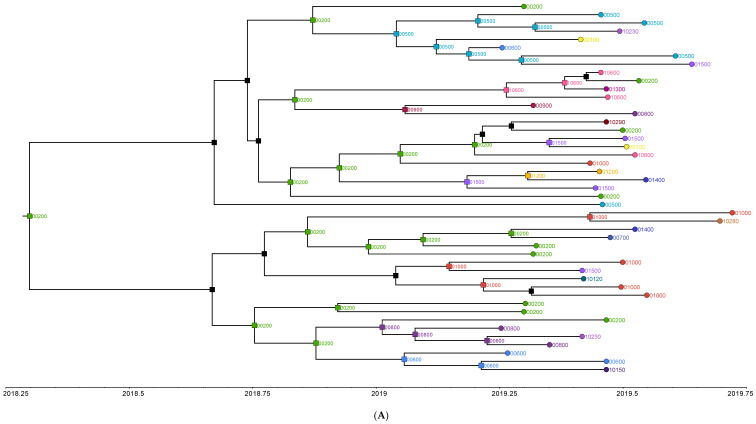

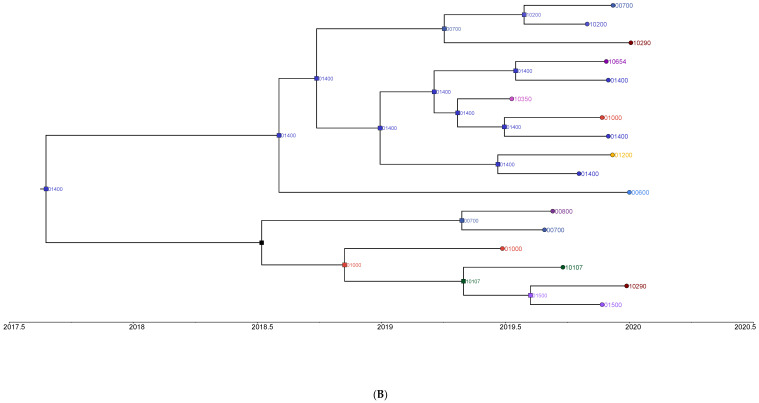

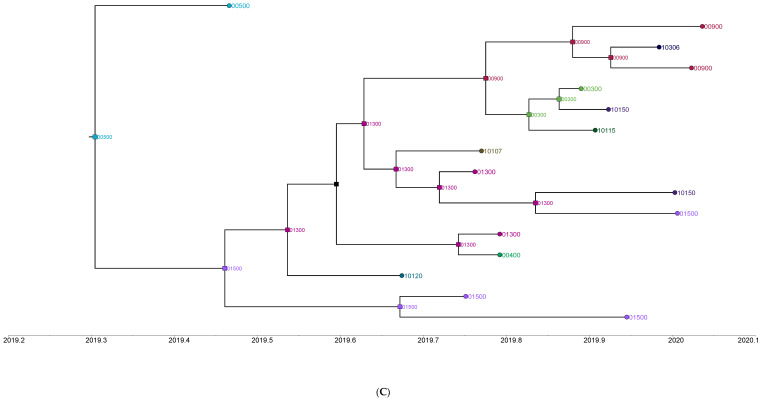

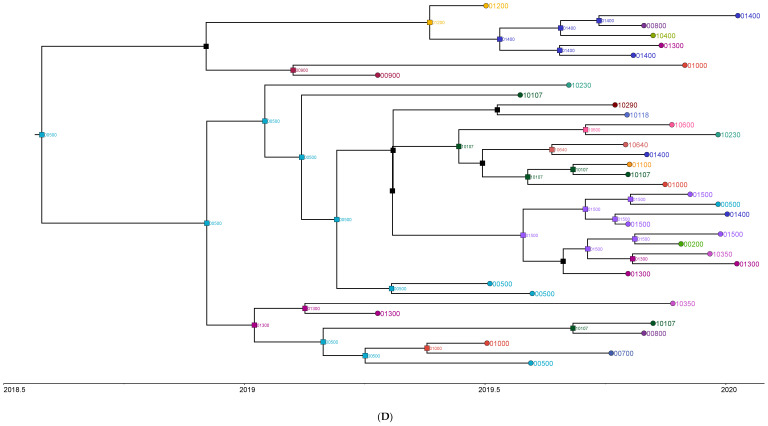
Maximum Clade Credibility (MCC) trees generated from (**A**) Cluster Q (DENV2 cosmopolitan sequences from April 2018 to September 2018), (**B**) Cluster R (DENV2 cosmopolitan sequences from April 2019 to January 2020), (**C**) Cluster S (DENV2 cosmopolitan sequences from April 2019 to January 2020), and (**D**) Cluster T (DENV3 genotype I sequences from April 2018 to September 2018). The branches are collapsed to show nodes with a posterior probability >0.5. The timeline at the bottom of each tree shows the time of existence of each extant sequence and their ancestors. The tips (circles) show postcodes of extant sequences, and nodes (squares) show imputed postcodes of ancestral sequences. Postcodes starting with 0 are within Colombo city and those starting with 1 are outside the city. The root of the whole tree indicates the most likely postcode of the most recent common ancestor.

When four outbreak clusters were analyzed with the phylogeographic methods described above, for all four clusters the imputed location of the most recent common ancestor was within the city limits (Figure 5A–D). These were postcodes 00200, 01400, 00500 and 00500 for clusters Q, R, S, T, respectively. In fact, out of 93 ancestral nodes, across all four clusters for which the location was imputed with a posterior probability >0.5, only 10 (10.7%) nodes had a postcode outside the Colombo city, despite 29% (33/114) of input sequences being from a postcode outside the city. The median distance between the imputed postcode at the root of the tree and those at the tips of the tree ranged from 3.49 km (IQR: 4.0 km) in cluster Q, to 6.06km (IQR: 5.12 km) in cluster T. The maximum distance between the imputed postcode at the root and at a tip of a tree was observed in cluster R, which was 18.5 km (Table 3).

### 3.4. Serotype Switches and Illness Severity

The DENV3 genotype I driving cluster T, from temporal outbreak bin 4 (April-2019 to January-2020), was previously seldom reported in Sri Lanka. However, the occurrence of plasma leakage, severe dengue, duration of illness and maximum symptom score within this cluster did not show a statistically significant difference (*p* > 0.05) when compared to that of cluster Q (DENV2 outbreak, from temporal bin 2) or clusters R and S combined (DENV2 outbreaks from temporal bin 4). All analyses were adjusted for previous dengue infection, which is a known confounder of disease severity. (Supplementary Table S1).

## 4. Discussion

This phylogenetic and phylogeographic analysis of DENV sequences isolated over a 28-month period between 2017–2020, from the Colombo district of Sri Lanka, showed that serotypes 1, 2 and 3 are transmitted in parallel within the community. DENV2 was the more dominant serotype in transmission until mid-2019, when a distinct DENV3 outbreak was noted. Most circulating viruses originated from relatively recent ancestors, although a few viral variants from plausibly earlier ancestors were also noted in circulation. There was no increase in disease severity in the outbreak associated with the most recently observed DENV3 outbreak, compared to the pre-existing DENV2 outbreaks. Outbreaks within the district were more likely to originate within the Colombo city and then spread to postcodes outside the city limits.

DENV sequencing data from Sri Lanka is scarce and mostly limited to partial genomes generated as part of research projects, because in-country sequencing facilities were not readily available prior to 2020. However, even this limited data confirms parallel circulation of multiple serotypes within the country. Secondary sources of the literature [32,33] suggest that DENV1 and 2 were first identified in Sri Lanka around 1965–1968. However, it was not possible to trace the original literature to confirm these claims. A more recent study that serotyped 605 dengue serum samples collected between 2003–2006 in the Colombo district, identified all four serotypes in circulation but found DENV2 and DENV3 were the dominant serotypes. These authors also used sequences generated from historical samples dating back to 1980s in their phylogeny and identified at least two genotypes of DENV1 (genotypes III and IV), a single genotype of DENV2, and mostly, discovered genotype III of DENV3 was in circulation from the early 1980s to 2006 in Sri Lanka. However, the data was limited to a few sequences, sometimes isolated years apart for the period prior to 2003. Furthermore, the analysis only used short segments of genomes (238–966 nts) in phylogenetic analyses and used an older version of dengue genotyping [34]. We have since shown that whole genomes are superior in phylogenetic analysis, compared to smaller genomic fragments, as it improves the resolution to infer phylogenetic relationships [23]. 

Despite the endemicity of all dengue serotypes, distinct outbreaks in recent history have been dominated by one serotype. For example, in 2009–2010, an outbreak of unprecedented magnitude was reported in Sri Lanka and whole genome sequencing of samples isolated from the Colombo district showed the dominant serotype had shifted from DENV2/3 to DENV1. Furthermore, the outbreak was driven by DENV1 genotype I, a genotype that was not previously reported in Sri Lanka [35]. A second large outbreak, which even surpassed the magnitude of the 2009/10 outbreak, occurred in 2017. Limited sequencing of the virus, mostly from the Colombo district, showed that the predominance of the circulating serotype had shifted back to DENV2 (cosmopolitan genotype) [13]. This finding was confirmed in our study because from late 2017 until mid-2019 the dominant circulating genotype was the cosmopolitan genotype of DENV2. From mid-2019, another outbreak, driven by DENV3 genotype I, seems to have occurred. Only two DENV3 genotype I sequences have been isolated from Sri Lankan samples, once in 2004 and later in 2017 prior to this study (https://legacy.viprbrc.org/, accessed on 20 April 2023) [36], and otherwise, almost all DENV3 sequences isolated from Sri Lanka belonged to genotype III. Regarding DENV1, two genotypes were noted in our study, but they could not be linked to outbreaks, and hence represent non-dominant endemic transmission. 

Interpretations of the timed Bayesian MCC trees in this paper are deliberately restricted to recent ancestral nodes representing diverging lineages within the same genotype. Other ancestral nodes further back in the timeline, representing ancestors between genotypes or serotypes, are more suitable for studying the broader evolution of the virus, which is beyond the scope of this paper. In this context, the timed phylogenies generated by BEAST revealed that the majority of currently circulating DENV2 sequences in CDS originated from an ancestor that existed around 2010, while most of DENV3/DENV1 sequences also originated from a more recent ancestor around 2015/2016. This shows that though transmission of all three serotypes is endemic, virulent strains can sweep the viral transmission landscape to provide rise to a majority of lineages currently in circulation. It is unclear how such virulent strains emerge but due to the discovery of genotypes previously unreported in Sri Lanka driving outbreaks in 2009 and 2019, imported infections is a possibility. Interestingly, the DENV3 outbreak in 2019 did not cause more severe clinical disease compared to the parallel DENV2 outbreak, probably because multiple parallelly circulating serotypes confer effective immunity via past infections against multiple serotypes within the community. It should be noted that CDS only recruits adult patients who are likely to have prior multiple infections (hence, immunity) and findings in children may be different [37].

Apart from the periodic “unusual” outbreaks as those reported in 2009 (DENV1), 2017 (DENV2) and 2019 (DENV3), seasonal outbreaks of a lesser magnitude (approximately two per year) driven by monsoon rains, have been a regular occurrence in the past decade in the Colombo district. In a two-step approach, as described above, we epidemiologically and phylogenetically identified sequences that can be linked to such outbreaks to perform phylogeography analyses to identify postcodes triggering such outbreaks. Out of all four, such clusters were identified; the ancestral event was within a postcode of the Colombo city (00100–01500). This highly urbanized area spanning over 37 km^2^ has the highest population density in the country. Thus, more concentrated vector elimination efforts within Colombo city, at least 2–4 weeks prior to the start of usually predictable seasonal outbreaks, may help to mitigate the impact of each outbreak within the entire Colombo district. 

The median distance of spread of related infections, in each of the clusters included in the phylogeography analyses, were in the range of 3.5–6.0 km, which is at least 10–20-fold higher than the average flight distance of the vector mosquito. This shows the importance of human (host) movement for outbreak spread over that of the vector. In addition to the resident population, many children and adults move in and out of the Colombo city limits daily for education and work. Traffic data suggests that up to 60,000 vehicles including public transport vehicles enter the city limits daily [38]. It is estimated that the daytime inflow into Colombo city is about one million people [39]. Thus, dengue infection, which has a longer asymptomatic incubation period of up to 2 weeks and then a short-lasting symptomatic illness (around 1 week), can spread rapidly through workplaces and schools, as people from multiple postcodes within and outside the district share close confined spaces within these institutions. The vector mosquito also prefers indoor environments (endophilic) and bites during the daytime [40], and is ubiquitously present throughout the district [41], and with all this considered, thus provide the ideal conditions for spread through asymptomatic viremic hosts.

This study has several limitations. Firstly, the observations are limited to the Colombo district of Sri Lanka, and hence, some findings (e.g., dominant circulating serotypes and genotypes) cannot be generalized to the whole country. However, there are many temporary residents within the Colombo district who return home to other districts during holidays or weekends, and aid disease transmission to other parts of the country. Given the relatively small size of the country, and that vector mosquitoes are universally prevalent, the patterns observed in the Colombo district may also be true for the rest of the country. Secondly, the phylogenetic and phylogeographic analyses are biased to available (extant) sequences. However, given our systematic continuous sampling from the same location over a 28-month period, which also happens to be the main teaching hospital in the district (and in the country), it can be reasonably concluded that the sequenced viral genomes are representative of currently circulating sequences within the Colombo district. Thirdly, in the phylogeographic analysis, a two-step process was used to reliably identify sequences linked to a specific outbreak event. This specificity was a trade-off against the number of sequences available per cluster, and hence, the representativeness of sequences from all available postcodes in the district. Therefore, it is not advisable to consider the specific postcode imputed for the most recent common ancestor as being the definite starting point of an outbreak. Similarly, the imputed postcodes for each intermediary ancestor within a lineage should not be interpreted as the definite route for infection spread. Fourthly, as the hospital was located within Colombo city (postcodes 00100–01500), patients from city postcodes were more likely to come to this hospital than those outside of the city limits. In each of the phylogeographic clusters, 60–80% of sequences came from the city postcodes; hence, the ancestral postcode at the root of the tree has a greater probability of being a city postcode. Yet, due to high urbanization and population density, the actual disease incidence is also higher within the city limits compared to that outside [36], and therefore, some part of this discrepancy is a true reflection of geographical disease incidence distribution. Finally, the location of infection may not be accurately reflected by the residential postal code as many people move in and out of the city each day, and centers of employment or learning may be a key contributor for infection that is not investigated in this study.

In conclusion, this 28-month long phylogenetic study within the Colombo district in Sri Lanka, using near-full length genomes, showed DENV1, DENV2 and DENV3 to be in circulation simultaneously. Throughout the observation period, most seasonal outbreaks were driven by the DENV2 cosmopolitan genotype, but since mid-2019, an outbreak of disproportionate magnitude was likely triggered by genotype I of DENV3, previously infrequently reported in Sri Lanka. However, this serotype switch was not accompanied by a change in clinical severity of disease, probably due to immunity against multiple serotypes among adults of the community. Phylogeographic analysis of sequence clusters showed that each outbreak is likely to have started within Colombo city and then spread throughout the district driven by both host and vector movement.

## Figures and Tables

**Table 1:** Serotype and genotype breakdown of DENV sequences analyzed in this study.

	DENV1		DENV2		DENV3		Total
	Genotype I	Uncategorized	Cosmopolitan	Other	Genotype I	Genotype III	
All sequences	49		223		73		345
Sequences that could be genotyped	7	36	216	3	65	1	328
Genotyped sequences from residents in Colombo district	4	22	148	2	51	1	228

**Table 2:** The summary of clusters selected for phylogeographic analysis.

Cluster Name	Serotype	Genotype	Bin	Number of Sequences
Q	DENV2	Cosmopolitan	2	45
R	DENV2	Cosmopolitan	4	17
S	DENV2	Cosmopolitan	4	16
T	DENV3	Genotype I	4	37

**Table 3:** Summary of the distances between the imputed postcode of the most common recent ancestor and that of extant sequences in each outbreak cluster.

Cluster Name	Root Post Code	Median (km)	IQR (km)	Max (km)
Q	00200	3.49	4.00	16.62
R	01400	4.71	9.91	18.57
S	00500	5.97	6.05	14.96
T	00500	6.06	5.12	12.26

## Data Availability

Viral sequence data will be uploaded to Genbank and linked to this paper once accession numbers are received. De-identified clinical data can be provided on request.

## Supplementary Materials

The following supporting information can be downloaded at: https://www.mdpi.com/article/10.3390/v15071408/s1, Supplementary Figure S1: Consensus Maximum Likelihood phylogenetic tree of all CDS sequences generated with RAxML with 1000 bootstraps and >70% consensus at each node; Supplementary Figure S2A: Maximum Clade Credibility tree of DENV2 temporal outbreak bin 1; Supplementary Figure S2B: Maximum Clade Credibility tree of DENV2 bin 2; Supplementary Figure S2C: Maximum Clade Credibility tree of DENV2 bin 3; Supplementary Figure S2D: Maximum Clade Credibility tree of DENV2 bin 4; Supplementary Figure S3: DENV3 genotype I sequences with >1000 nt and CDS DENV3 genotype I sequences were used to generate the Maximum Likelihood tree using RAxML with 1000 bootstraps and >70% consensus at each node, and close up view of the CDS sequences and their closely related sequences; Supplementary Table S1: Summary statistics of each of the clusters.
