# Supplementary material for: Genomic Surveillance of Recent Dengue Outbreaks in Colombo, Sri Lanka
Source: Viruses. 2023 Jun 21;15(7):1408. doi: 10.3390/v15071408 (PMC10384240; doi:10.3390/v15071408)

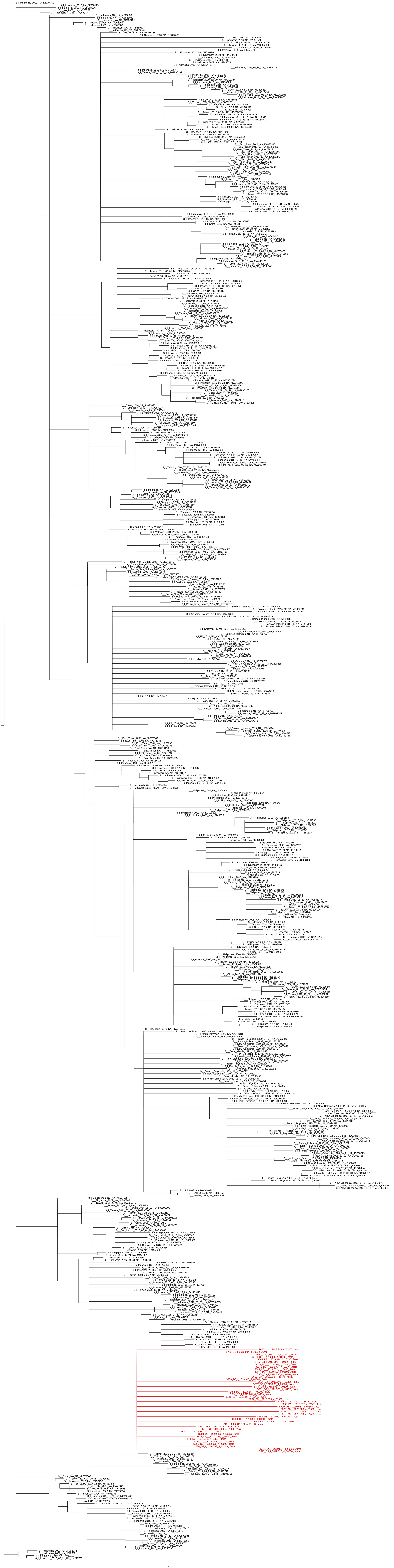

Supplement: Supplementary file 1 [file viruses-15-01408-s001.zip › Supplementary Figure S3.pdf]
