# Supplementary material for: Genomic Surveillance of Recent Dengue Outbreaks in Colombo, Sri Lanka
Source: Viruses. 2023 Jun 21;15(7):1408. doi: 10.3390/v15071408 (PMC10384240; doi:10.3390/v15071408)
Supplement: Supplementary file 1 [file viruses-15-01408-s001.zip › viruses-2438558-Table S1.pdf]

Supplementary Table 1

Summary of illness severity statistics of each of the sequence clusters used in phylogeography analysis.

A symptom score was calculated each day of hospitalization and the maximum value was selected for analysis. P>0.05 for all comparisons when adjusted for previous dengue infections.

Cluster	Cases	Maximum symptom score (Mean/SD)	Duration of hospitalization (Mean/SD)	Severe dengue (valid percent)	Plasma leakage (valid percent)
Q	45	4.36 /1.464	6.284 /1.8097	4.4	55.6
R	17	4.58 /2.021	5.8 /1.5586	0	75
S	16	4.23 /1.6141	5.668 /1.7523	7.7	69.2
T	37	4.68 /1.62	6.756 /1.2869	3.2	64.5
