# Supplementary figures and images for: Genomic Surveillance of Recent Dengue Outbreaks in Colombo, Sri Lanka
Source: Viruses. 2023 Jun 21;15(7):1408. doi: 10.3390/v15071408 (PMC10384240; doi:10.3390/v15071408)

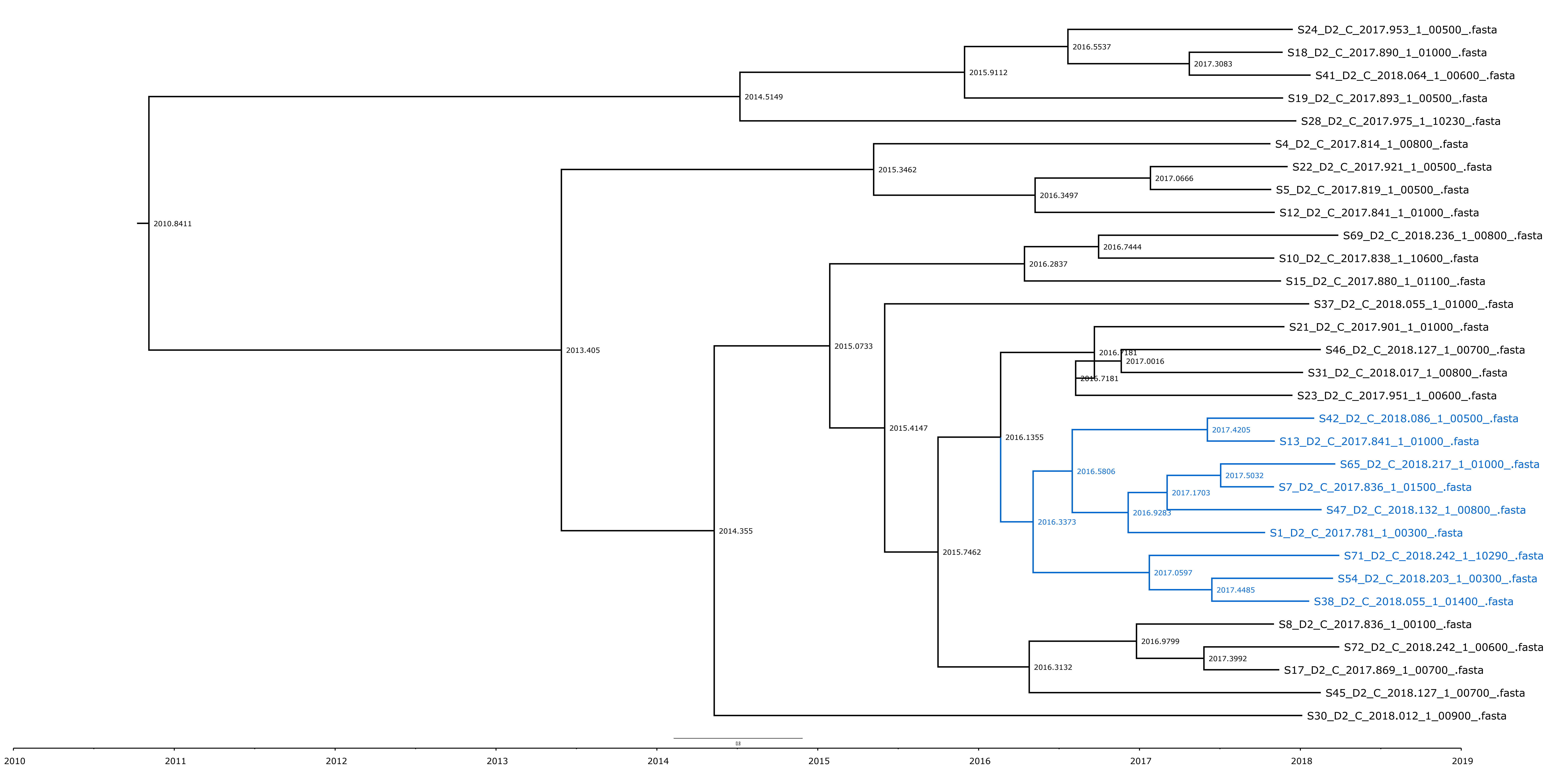

Supplement: Supplementary file 1 [file viruses-15-01408-s001.zip › Supplementary Figure S2A.pdf]

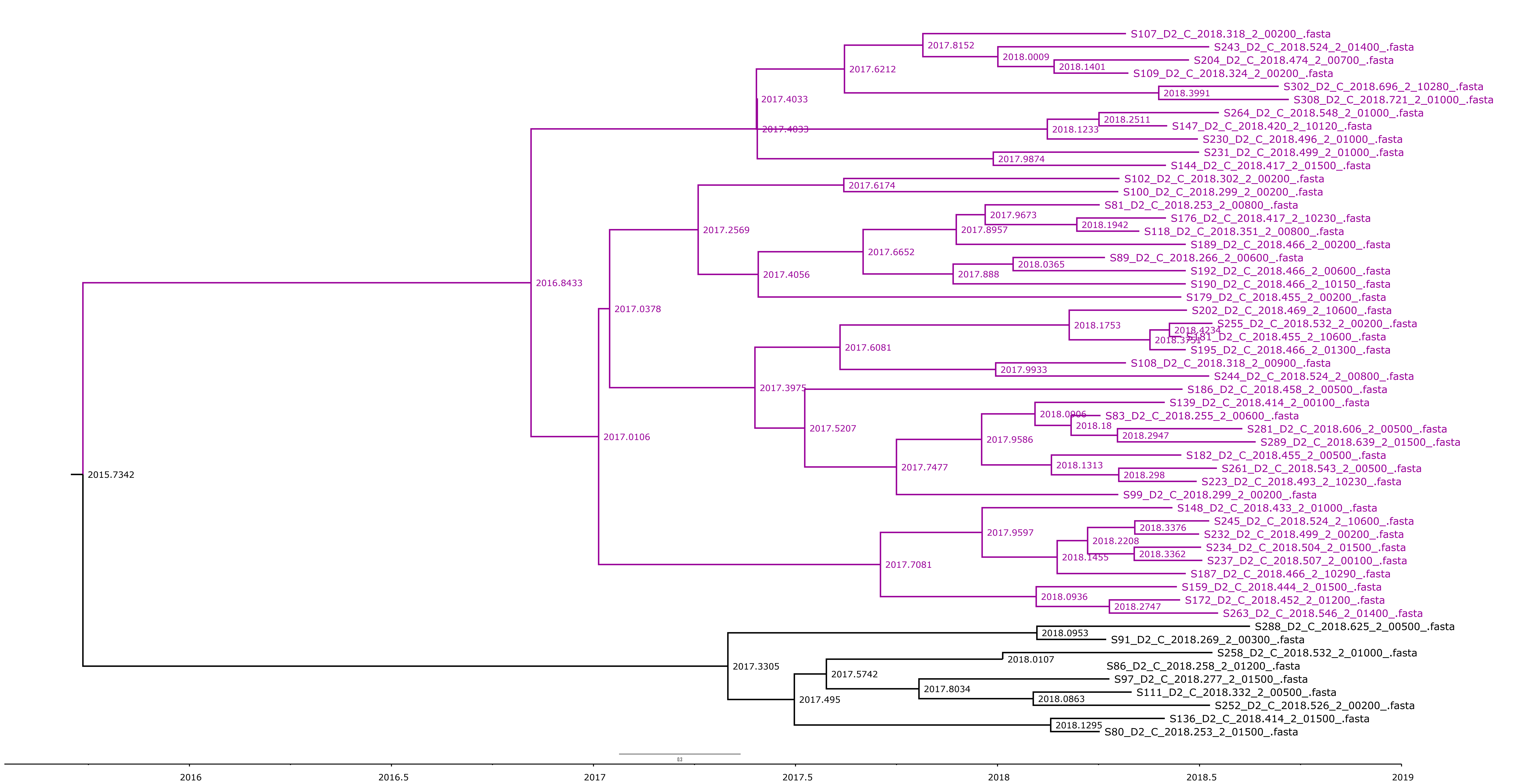

Supplement: Supplementary file 1 [file viruses-15-01408-s001.zip › Supplementary Figure S2B.pdf]

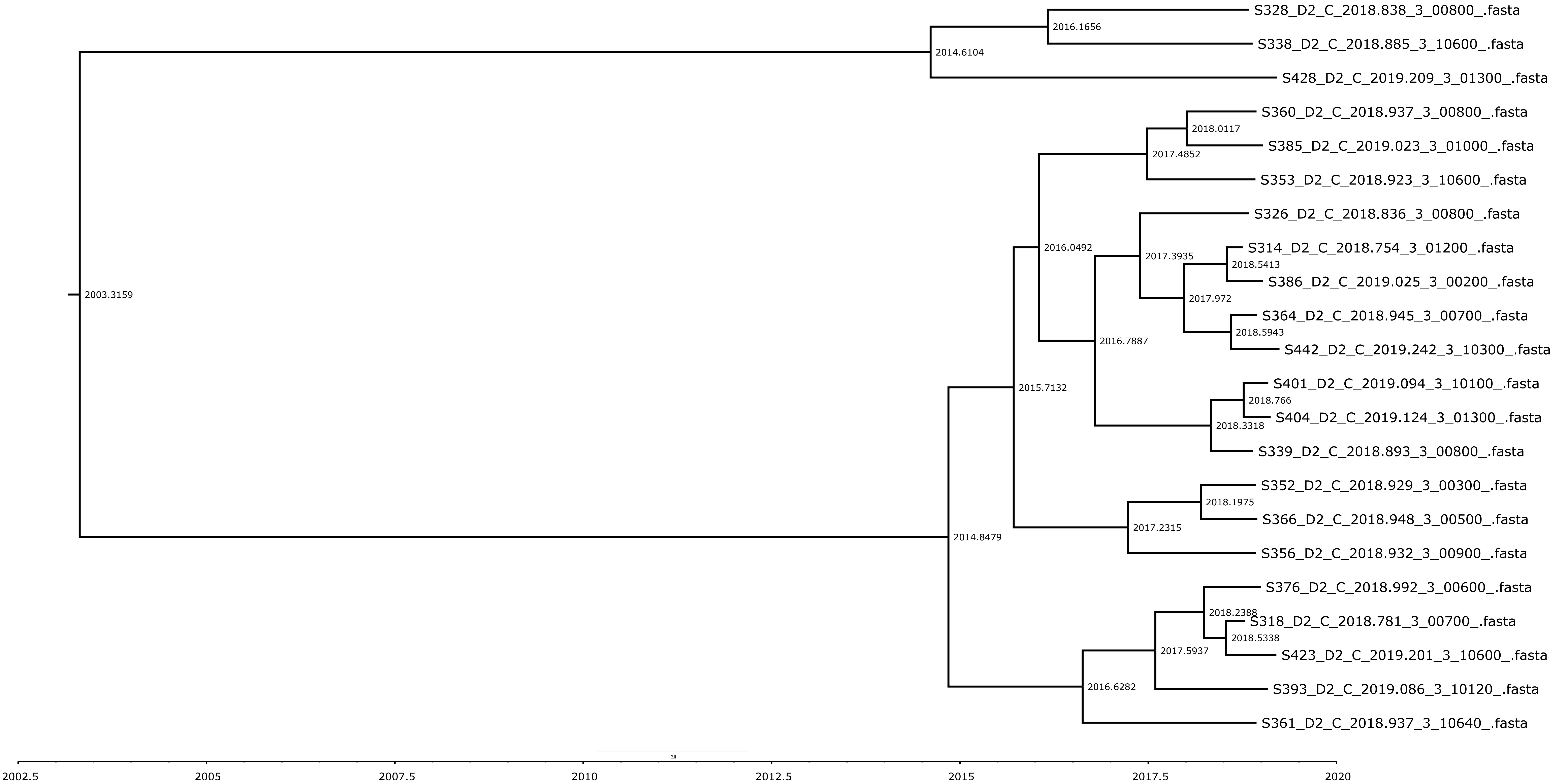

Supplement: Supplementary file 1 [file viruses-15-01408-s001.zip › Supplementary Figure S2C.pdf]

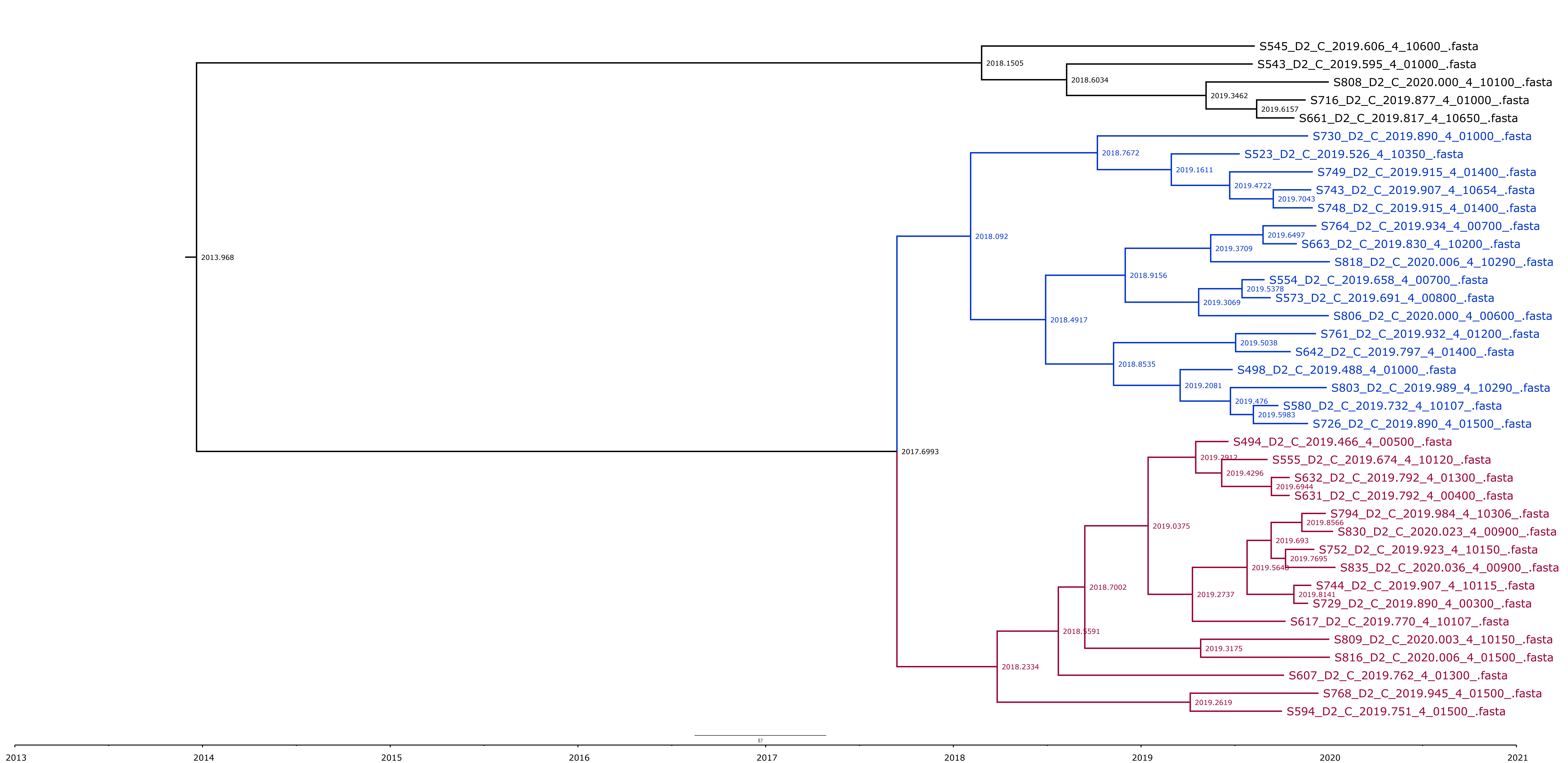

Supplement: Supplementary file 1 [file viruses-15-01408-s001.zip › Supplementary Figure S2D.pdf]

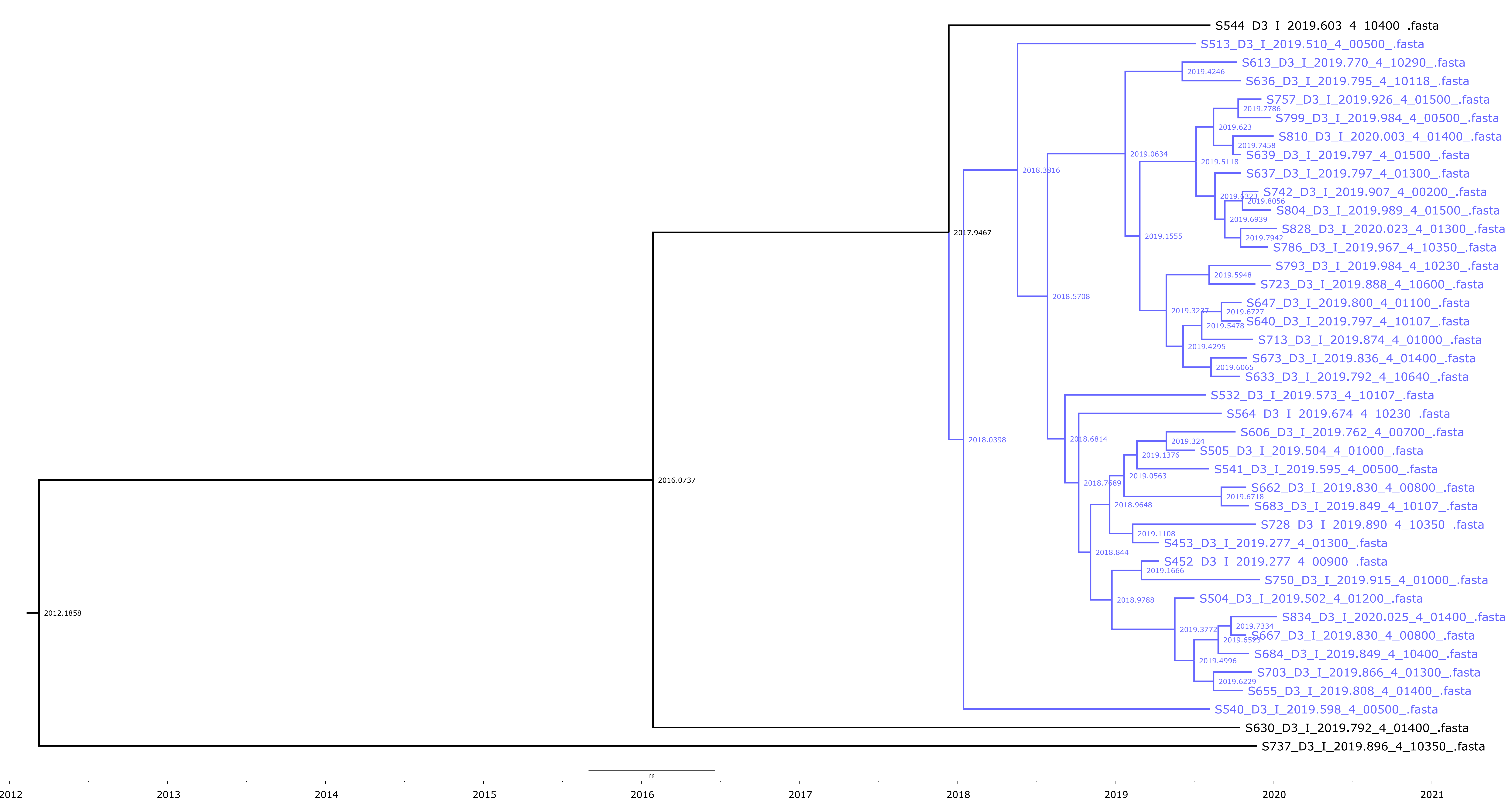

Supplement: Supplementary file 1 [file viruses-15-01408-s001.zip › Supplementary Figure S2E.pdf]
